# Terminal ileal trichophytobezoar with perforation: A rare presentation with review

**DOI:** 10.4103/0971-9261.42575

**Published:** 2008

**Authors:** Ronald Scorpio, Devesh Sharma

**Affiliations:** Division of Pediatric Surgery, Geisinger Medical Center, 100 North Academy Ave, Danville, Pennsylvania, USA

**Keywords:** Intestinal, perforation, trichophytobezoar

## Abstract

Trichophytobezoars are uncommon foreign bodies, formed by hairs and vegetable matter with impacted food particles. We report an unusual case of a 5-year-old girl with large terminal ileal trichophytobezoar causing perforation peritonitis.

## INTRODUCTION

Trichobezoar is derived from trichos (hair) and bezoar (foreign body). The word bezoar is derived from the Arabic “badzehr” or from the Persian padzahr and it means antidote. Trichophytobezoar is composed of mixed hairs and indigestible vegetable fiber content. Other contents of non-biological origin (Iniobezoar) e.g, medications, foreign bodies such as coins, buttons, screws, parts of toys, bubble gum, etc. have also been described.[1,2]

Clinically trichophytobezoars are found most commonly in stomach. We present a case report of a trichophytobezoar found in terminal ileum with a tail extending distally into cecum and ascending colon, causing a large perforation with perforation peritonitis. After evaluating the bezoar post-operatively, it appeared to be a very rare case of a gastric bezoar with tail (Rapunzel syndrome), passing off the pylorus in toto and causing small bowel obstruction distally.

## CASE REPORT

**Figure 1:**
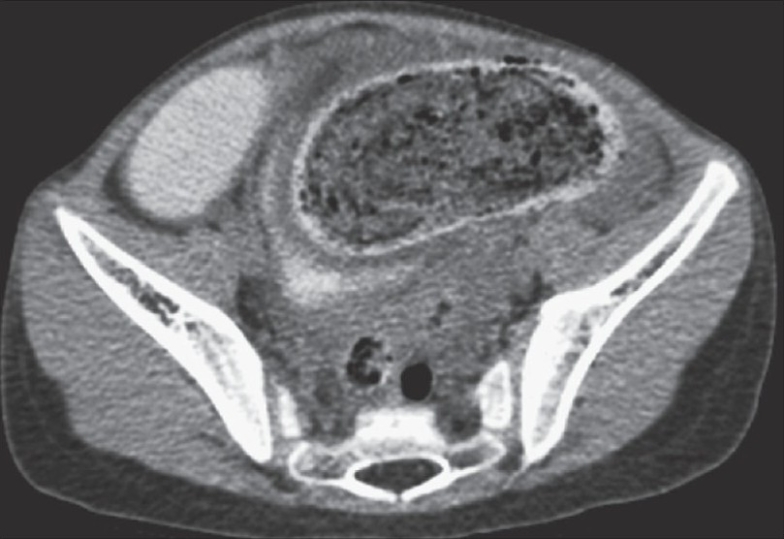
Extraluminal air with bezoar

**Figure 2:**
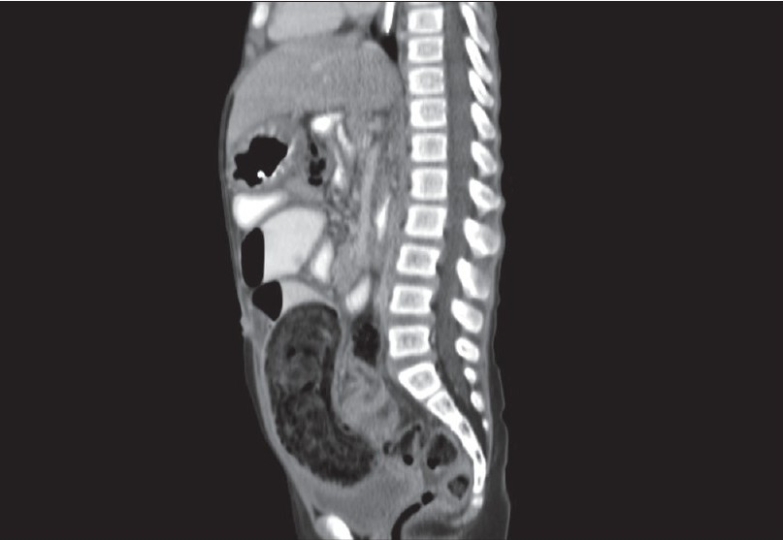
Sagittal view on CT with bezoar

**Figure 3:**
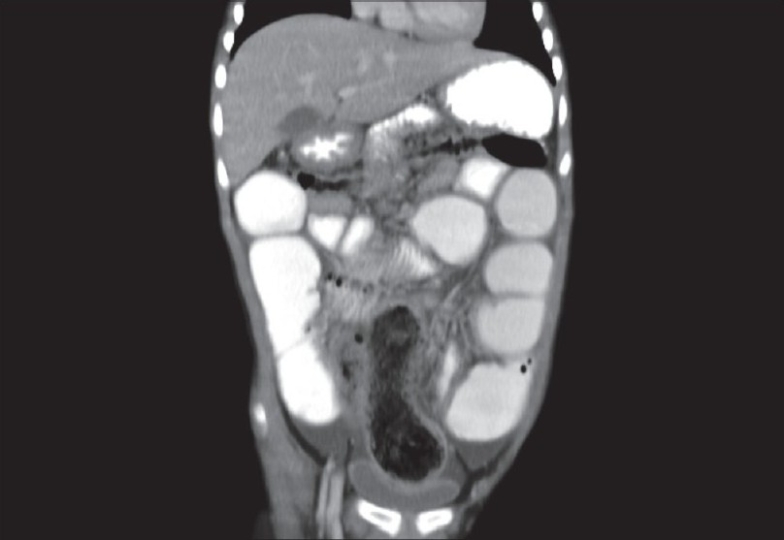
Coronal view on CT with bezoar

A 5-year-old girl was admitted with the complaints of chronic constipation with abdominal pain. Patient was a thin but otherwise healthy looking girl. She was admitted 3 months earlier with similar complaints and was treated conservatively with intravenous hydration. A plain abdominal X-ray showed dilated small bowel loops with no free air. There were no signs of generalized peritonitis. After initial resuscitation, a CT scan with oral and intravenous contrast was done. CT scan was suggestive of small extra-luminal air bubbles on the outside margin of a distal small bowel loop [Figure 1]. Also a suspicion was raised about the presence of a large bezoar contained in the distal small bowel or in the cecum [Figures 2 and 3]. With the diagnosis of perforation peritonitis with free intra-abdominal air, the patient was taken for emergency operation.

**Figure 4:**
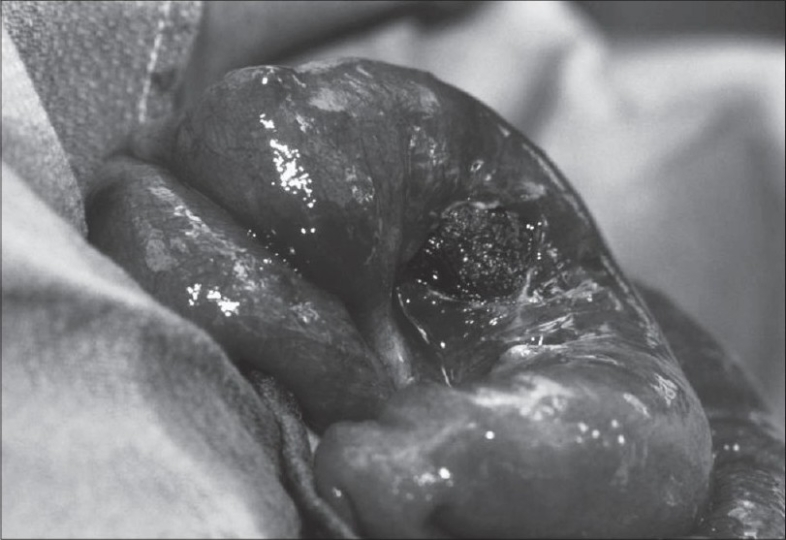
Perforation in terminal ileum, bezoar can be seen through the perforation

**Figure 5:**
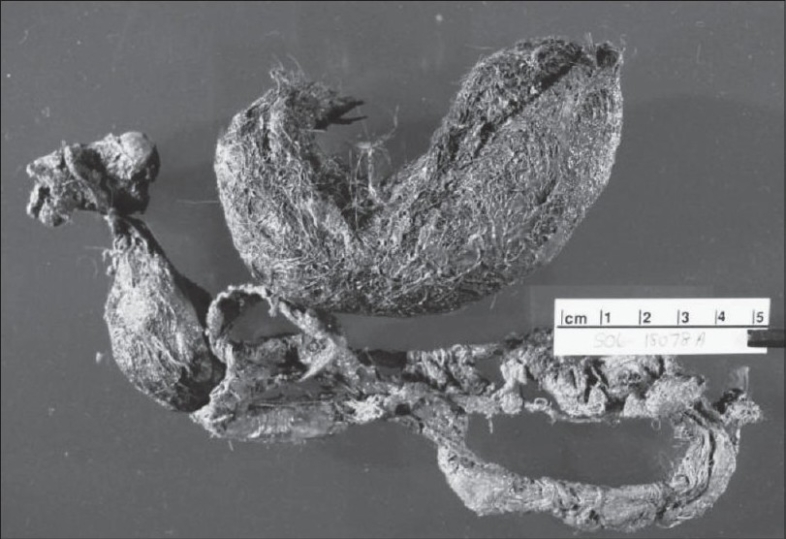
Trichophytobezoar with divided tail

An exploration laparotomy was performed through the right lower quadrant transverse incision. A large trichophytobezoar was identified through the perforated terminal ileum [Figure 4]. Perforation was present 8 inches proximal to ileocecal junction. With the diagnosis of trichophytobezoar causing the perforation and the proximal obstruction, decision was made to excise the involved bowel segment. A long tail of the bezoar was identified. Distal stapled segment of the ileum was opened and tail of the bezoar extending into the cecum and right colon was removed. No proximal tail suggesting a proximal bezoar was identified after opening the proximal loop of ileum. Appendectomy was performed prior to the closure of the incision to avoid confusion about the diagnosis of appendicitis in future. When the small bowel specimen was opened postoperatively, bezoar appeared like a gastric bezoar with a long tail (Rapunzel syndrome) [Figure 5]. Patient was discharged to home on the fifth postoperative day. She was readmitted on the seventh postoperative day for minor wound infection and was discharged 3 days later in good health. She is doing well after 6 months follow-up. Post operative psychiatric evaluation showed that the patient was psychologically stable and did not reveal any evidence of anxiety or depression.

## DISCUSSION

In the pediatric population, the common types of bezoars are lactobezoars, trichobezoars, phytobezoars and foreign bodies. The first description of bezoars, as a human disease was done by the Frenchman Baudament in 1779. The removal of bezoars surgically, was first carried out by Schonborn in 1883.[3]

In the pediatric population, trichobezoars are seen most commonly in females. According to the review by DeBakey and Ochsner, 18% of cases occurred during the first decade of life, 37% during the second and 27% in the third. The earliest occurrence was reported by Gaston in a one-year-old infant.[4] Incidence of trichotillomania has been described as 1 in 2000 children but trichophagia is seldom seen in these children. Also a bezoar does not form in all children with trichophagia.[5]

Trichobezoar commonly forms in the stomach. Trichobezoar from stomach can pass into small intestine in three ways,[1] when fragments of gastric trichobezoar break and pass through pylorus into the small bowel,[2] when complete bezoar passes off the pylorus in toto and gets stuck in the distal small bowel or[3] when strings of hairs pass through the pylorus in continuity with the main gastric bezoar, leading to famous Rapunzel syndrome. Rao *et al.* separated the complications associated with bezoars into mechanical and traumatic categories, the former incorporating gastric outlet and intestinal obstruction and the later necrosis, ulceration, hemorrhage and perforation.[6] Other complication including acute pancreatitis (secondary to irritation and edema of ampulla of Vater), appendicitis, obstructive jaundice, protein losing enteropathy and vitamin B12 deficiency have also been described.[7]

The recommended treatment for large or complicated trichophytobezoars is surgery. Depending on the presentation, surgery can vary from simple gastrotomy and removal of bezoar to intestinal resection for multiple or large perforations. Gastrotomy is not indicated in all cases. If patient presents with small bowel obstruction, enterotomy may be required instead.[8] Endoscopic removal after enzymatic dissolution of small bezoar may be attempted but carries the risks of perforation of the ulcer and metabolic acidosis. Close psychiatric follow-up is recommended to diminish the chances of recurrence.
